# Unsuspected paraganglioma of the urinary bladder with intraoperative hypertensive crises: A case report

**DOI:** 10.3892/etm.2013.1242

**Published:** 2013-08-01

**Authors:** SHIGUANG LI, SU LUI, FEI LI, QIANG YUE, XIAOQI HUANG, QIYONG GONG

**Affiliations:** 1 Department of Radiology, Huaxi MR Research Center (HMRRC), West China Hospital of Sichuan University, Chengdu, Sichuan 610041;; 2 Department of Radiology, The First People’s Hospital of Zunyi, Zunyi, Guizhou 653002, P.R. China

**Keywords:** paraganglioma, pheochromocytoma, urinary bladder, computed tomography

## Abstract

Paraganglioma of the urinary bladder is rare, accounting for <0.05% of all bladder tumors. Common clinical findings in patients with bladder paraganglioma include hematuria and intermittent hypertension during urination, along with generalized symptoms due to increased levels of catecholamines. Although unsuspected bladder paraganglioma may result in intraoperative hypertensive crises, these may be avoided if characteristic imaging signs are observed. The present study reports a case in which a patient with unsuspected paraganglioma experienced a severe hypertensive episode during cystoscopic tumor resection. Although this case had typical computed tomographic characteristics of the bladder paraganglioma, the possibility of the paraganglioma pre-operatively was not taken into account.

## Introduction

Bladder paraganglioma (pheochromocytoma) is rare, accounting for <0.05% of all bladder tumors and ∼10% of extra-adrenal paragangliomas (1). Hematuria and the symptoms induced by micturition or defecation due to an excess of catecholamines, including hypertension, palpitations and headache, are typical clinical findings in patients with bladder paragangliomas. Computed tomography (CT) and magnetic resonance imaging (MRI) may provide specific characteristic signs for the diagnosis of paraganglioma (2–4), particularly in cases where patients lack the typical symptoms. However, bladder paraganglioma is not considered as the primary pre-operative diagnosis for patients with bladder neoplasm, and these unsuspected paragangliomas greatly increase the peroperative mortality rate in patients undergoing even relatively minor surgical procedures (5). In the present study, a case of unsuspected bladder paraganglioma is reported, in which characteristic signs of the condition were exhibited on CT images, and a severe hypertensive episode occurred during cystoscopic tumor resection. The study was approved by the ethics committee of West China Hospital of Sichuan University, Chengdu, China. Written informed consent was obtained from the patient.

## Case report

**Figure 1.**
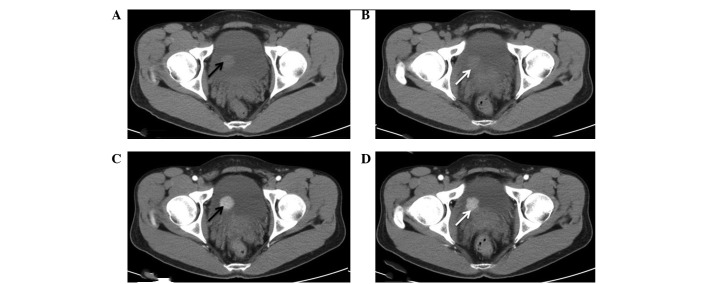
Computed tomography (CT) scanning revealed a 26×22×25 mm polypoid lesion protruding into the bladder cavity (black arrow) with a pedicle (white arrows) connected to the right postero-lateral wall of the urinary bladder, well-defined borders and no thickening of the wall near the tumor. (A and B) On the plain CT images, the mass demonstrated homogeneous iso-density and (C and D) on the contrast-enhanced CT images, homogeneously significant enhancement was observed. The degree of enhancement of the tumor was significantly higher than that of the bladder wall.

A 26-year-old male patient was admitted to West China Hospital of Sichuan University on August 15, 2012 with a history of a bladder tumor and microscopic hematuria. The lesion was found 2 years before by abdominal ultrasonography (USG) and the size of the tumor was stable from then until 4 months prior to admission, when the tumor began to enlarge. Physical examination of the patient did not reveal any unusual findings; the blood pressure of the patient was 93/63 mmHg on admission and results of the urinalysis were normal. An abdominal CT examination was performed using a Philips Brilliance 64-slice CT scanner (Philips Medical Systems, Eindhoven, The Netherlands) with a slice thickness of 6 mm. This revealed a 26×22×25 mm nodular lesion originating from the right bladder trigone region, protruding into the bladder cavity. The tumor showed homogeneous soft-tissue attenuation [mean CT value, 40 Hounsfield units (HU)] on a plain CT scan, the density of which was slightly lower than that of the internal obturator muscles. The lesion exhibited intense enhancement (mean CT value, 129 HU) in the arterial phase, which was significantly higher than that of the bladder wall and only slightly lower than that of the right femoral artery. Thickening of the bladder wall near the tumor was not observed (Fig. 1). The primary radiological and clinical diagnosis was bladder carcinoma with no suspicion of extra-adrenal paraganglioma.

Following the preoperative examinations, an elective cystoscopic tumor resection was performed on August 17, 2012. During the surgery, the tumor was found in the trigone region of the bladder. The tumor had a pedicle, smooth surface, edema mucosa and varicose veins. On attempted surgical removal of a small part of the tumor, the blood pressure of the patient suddenly increased to 210/120 mmHg and the heart rate reached 145 bpm. The operative procedure was immediately terminated, and the blood pressure and heart rate gradually returned to normal over 10 min. Subsequently, an intraoperative biopsy using frozen sections indicated the presence of paraganglioma.

**Figure 2.**
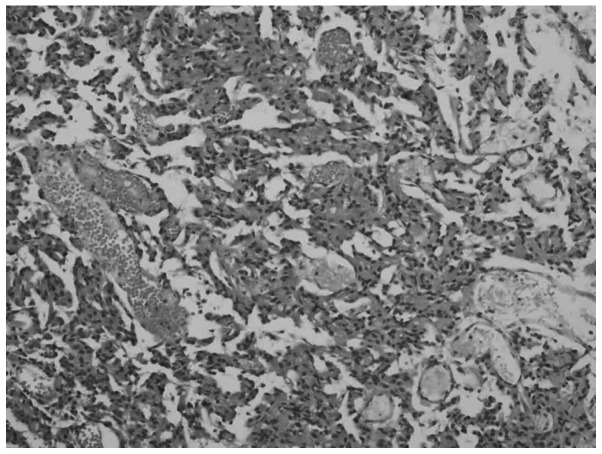
Microscopic examination of the resected tumor revealed that tumor cells were polygonal or round in shape, arranged in nests and rich in eosinophilic fine granular cytoplasm. Acinus-like structures were observed and the interstitial spaces were rich in capillaries (hematoxylin and eosin staining; magnification, ×200).

Following surgery, the history of the patient was reviewed and it was noted that the patient had a history of post-micturition syncope. One day after the surgery, the erect and supine aldosterone levels of the patient were 15.51 ng/dl (normal, 9.8–27.5 ng/dl) and 17.57 ng/dl (normal, 4.5–17.5 ng/dl), respectively), and the plasma renin levels were 1.59 ng/ml/h (normal, 0.56–2.79 ng/ml/h) and 1.65 ng/ml/h (normal, 0.05–0.8 ng/ml/h), respectively. Four days after the surgery, the plasma norepinephrine and epinephrine levels were 345 ng/l (normal, 272–559 ng/l) and 91 ng/l (normal, 54–122 ng/l), respectively. A partial cystectomy with complete extirpation was performed on August 28, 2012. During the second surgery, no episodes of hypertension occurred. Convalescence was uneventful. Histological examination confirmed bladder paraganglioma (Fig. 2).

## Discussion

Bladder paragangliomas may occur in patients of any age, but typically occur between the ages of thirty and fifty (6). They are usually submucosal or intramural with intact vesical epithelium and, consistent with the present case, are commonly detected in the trigone region (7).

The most common feature of bladder paraganglioma is a characteristic clinical picture resulting from a hypertensive crisis that may be accompanied by headache, palpitations, hot flushes and sweating. These crises are typically provoked by micturition or overdistention of the bladder. The classical triad of episodic hypertension, persistent hematuria and post-micturition syncope is almost diagnostic, but is extremely rare (8). In the present case, painless hematuria was the primary clinical symptom. This is common in bladder disease and therefore does not specifically indicate the presence of bladder paraganglioma. Symptoms resulting from hypertensive crises did not present in this case, with the exception of a history of post-micturition syncope. Although this is a virtually diagnostic symptom, it is often missed when the history of the patient is initially taken, as occurred in this case.

The imaging modalities for the diagnosis of bladder paraganglioma include USG, CT, MRI and meta-iodobenzylguanidine (MIBG) scintigraphy (3,4). The characteristic signs of bladder paragangliomas in CT and MRI images are as follows: i) The tumor is nodular or a mass with homogeneous or heterogeneous density/signal; ii) the lesion often originates from the bladder trigone and seldom from the anterior or lateral wall, with well-defined borders; and iii) the lesion appears mostly as soft-tissue attenuation on a plain CT scan (2,3) with a slightly higher signal intensity than that of the gluteal muscle on pre-enhanced T1-weighted imaging (T1WI), and hyperintensity is observed compared with the gluteal muscle on T2WI. Unlike adrenal paraganglioma, bladder paraganglioma does not exhibit the characteristic ‘light bulb bright signal’ in T2WI (2,4). Studies using CT and MRI report significant enhancement of tumors following contrast injection (2–4). Epithelial tumor is the main disease for differential diagnosis of the bladder paraganglioma, and has been described to have a similar or reduced density compared with that of the bladder wall on enhanced scans (9). Epithelial tumors also tend to be more infiltrative and are associated with thickening of the adjacent bladder wall contiguous with the mass. By contrast, paragangliomas are extremely vascular and consequently exhibit greater enhancement than that of the urinary bladder wall. They also tend to be more localized (10). Although these characteristic signs of bladder paraganglioma were exhibited in the present case, clinical misdiagnosis occurred prior to the operation, leading to an intraoperative hypertensive crisis. Two explanations may account for this clinical misdiagnosis. Firstly, typical clinical signs, with the exception of microscopic hematuria, were not observed in this patient. The history of post-micturition syncope was not revealed at the initial visit. Secondly, the radiologist did not raise the possibility of bladder paraganglioma. The localized tumor with significant enhancement on contrast enhanced CT images should prompt the radiologist to consider bladder paraganglioma and subsequently warn surgeons to prepare for an intraoperative hypertensive crisis. Therefore, the current case highlights important points to be considered upon diagnosis. Firstly, it is important to record a thorough history of patients with bladder tumors, and in particular, to screen for a history of hypertensive episodes. Secondly, in the case of localized bladder tumors with significantly higher enhancement than that of the bladder wall on the CT image, the possible diagnosis of bladder paraganglioma should be raised in order to ensure that surgeons and anesthesiologists are prepared for an intraoperative hypertensive crisis.
